# The Use of Telerehabilitation Among Libyan Physiotherapists During the COVID-19 Pandemic: A Cross-Sectional Study

**DOI:** 10.3390/ijerph22091414

**Published:** 2025-09-10

**Authors:** Sami Elmahgoub, Adel El Taguri, Amira Ben Said, Farah Abu Khadra, Aseel Aburub, Ákos Levente Tóth

**Affiliations:** 1 Department of Physiotherapy, Faculty of Allied Medical Sciences, Applied Science Private University, Amman 11931, Jordan; a_abualrob@asu.edu.jo; 2 Department of Community Medicine, Faculty of Medicine, University of Tripoli, Tripoli 13932, Libya; tajoury@doctor.com; 3 Department of Physiotherapy, Faculty of Medical Technology, University of Tripoli, Tripoli 13932, Libya; waycool400@yahoo.com; 4 Doctoral School of Health Sciences, University of Pécs, 7621 Pécs, Hungary; a014bk@pte.hu; 5 Institute of Sport Sciences and Physical Education, Faculty of Sciences, University of Pécs, 7624 Pécs, Hungary; tothahu@gmail.com

**Keywords:** COVID-19, physiotherapy, telehealth, knowledge

## Abstract

**Background**: The COVID-19 pandemic has significantly disrupted healthcare delivery globally, particularly affecting physiotherapy practices that rely on close patient interactions. **Objectives**: This study investigates the knowledge and readiness of Libyan physiotherapists to adopt telerehabilitation during the pandemic. **Methodology**: A cross-sectional questionnaire-based study was conducted on a total of 109 physiotherapists who were recruited through convenience sampling from public and private hospitals, with a demographic distribution of 54 males and 55 females, aged 25 to 55 years. **Results**: Approximately 64% of physiotherapists reported being impacted by COVID-19. Among the participants, 67.9% indicated they had limited knowledge of telerehabilitation, whereas only 32.1% were familiar with the concept. Of those who were knowledgeable, only 57.1% had actually practiced telerehabilitation, and just 35% successfully integrated it into their patient management strategies. Key barriers to implementation included poor internet connectivity (71.6%) and high technology costs (38.5%). **Conclusions**: Our study highlights the importance of telerehabilitation for the future of physical therapy, particularly for patients with chronic conditions. Proper staff training, robust IT infrastructure, and patient education are all essential for enhancing the quality of service physiotherapists deliver in today’s evolving healthcare landscape.

## 1. Introduction

Since the global spread of the COVID-19 pandemic in 2019 and the subsequent declaration of a state of emergency, governments and health authorities have implemented various protective measures to curb the virus’s transmission. Many countries enforced strict isolation protocols and advocated for social distancing, leading to the closure of recreational activities and significant disruptions to daily life [1,2]. While these measures are crucial for public health, they have had a profoundly impactful effect on healthcare providers, particularly physiotherapists, who typically engage in close contact with patients [3,4]. The mandated social distancing requiring at least six feet between individuals has rendered many physiotherapy services inaccessible due to shelter-in-place orders.

This unprecedented situation has heightened the challenges and opportunities within digital healthcare practices. The rapid growth of digital technology in healthcare has introduced a variety of related terms that highlight different aspects of remote care, such as telehealth, telemedicine, and telerehabilitation. Each term focuses on unique aspects of delivering healthcare remotely. Telehealth is the broadest concept, covering everything from clinical services to patient education and administrative tasks, all delivered remotely [5]. Zooming in, telemedicine focuses specifically on the practice of medicine, allowing doctors to diagnose and treat patients from a distance [6]. The telerehabilitation takes things a step further by catering specifically to rehabilitation needs. This means that physical therapists and other specialists can guide patients through their recovery journeys without needing to be in the same room [7]. Understanding these differences is key as we explore the exciting opportunities and challenges that come with these new ways of providing care.

Many physiotherapists have found themselves lacking the necessary competencies for telerehabilitation implementation. Consequently, physiotherapy professionals need to consider key recommendations for delivering safe and effective digital services. This highlights the importance of physiotherapists adapting their practices to prioritize patient safety and ensure they receive the best possible care. Patients have been advised to avoid medical centers that may pose infection risks, which lead to delays in non-urgent care. However, for patients whose needs cannot be postponed, telemedicine has emerged as a viable alternative during quarantine and resource-limited conditions [8]. Over the past decade, healthcare providers have been gradually adopting telemedicine [9], but the COVID-19 pandemic necessitated a rapid shift toward remote consultations.

The pandemic has compelled the physiotherapy profession to explore digital solutions as a means of maintaining service accessibility [10,11]. World Physical Therapy and numerous national organizations advocated for remote rehabilitation to enhance accessibility [12] and provided resources and guidance for implementing services during the pandemic [13]. Studies have highlighted clinicians’ perspectives and acceptance of telerehabilitation, revealing high satisfaction rates among healthcare providers [12,14]. These positive impressions stem from advantages such as increased work flexibility, reduced space constraints in healthcare facilities, cost-effectiveness, and decreased travel time for patients [15,16].

As we move beyond the pandemic, it is crucial to evaluate the effectiveness of telerehabilitation as a component of physiotherapy practice. Understanding the comparative effectiveness of telerehabilitation versus traditional methods will help to determine its role, whether as a viable alternative or a complementary service to in-person physiotherapy. Research indicates that telerehabilitation can enhance physiotherapists’ use of technology. For instance, a study by Cottrell et al. demonstrated that clinicians improved their understanding of patient needs in a spinal care setting through telerehabilitation, despite the inherent challenges of maintaining physical contact [17]. While sophisticated telerehabilitation systems, like the Australian eHAB™ program, combine real-time videoconferencing with effective remote diagnosis, an adequate alternative for hands-on interventions remains elusive [18]. However, barriers to implementing telerehabilitation include inadequate infrastructure, such as poor internet coverage, and a lack of necessary telecommunication devices [16,17]. Furthermore, concerns regarding patient safety and privacy also hinder the adoption of telerehabilitation [11,17,19]. Regulatory and legal restrictions further complicate online consultations, discouraging physiotherapists from utilizing these services [20,21]. Previous studies have identified these challenges and urged governments to create pragmatic policies to promote telerehabilitation [21,22,23,24]. Understanding healthcare providers’ perspectives on implementing telerehabilitation is critical for its sustained adoption [19].

The COVID-19 pandemic has accelerated the adoption of telerehabilitation all over the world. For instance, countries like Australia and the United States have seen significant increases in telerehabilitation usage among physiotherapists, with studies indicating that up to 84% of practitioners utilized telerehabilitation services during the pandemic [25,26]. However, the effectiveness and acceptance of these services vary widely depending on each country’s healthcare infrastructure and technological readiness. In contrast, Libya’s healthcare system is state-funded, primarily organized under the Ministry of Health, and free at the point of service. The Ministry of Health oversees public hospitals and clinics, while private healthcare facilities also play a significant role. There are significant disparities between urban and rural areas. However, the ongoing conflict has damaged infrastructure, caused shortages of medical supplies and personnel [27]. Additionally, the regulatory frameworks for telehealth are still underdeveloped, which complicates the integration of digital health solutions into routine practice [28]. Limited internet connectivity and the high costs of technology further exacerbate these challenges, making it difficult for healthcare personnel to utilize telehealth effectively [28]. These issues hinder telehealth adoption, and by addressing these barriers is crucial for improving healthcare delivery and ensuring that physiotherapy services remain accessible to patients across Libya.

Although many studies have looked at how telerehabilitation has been adopted around the world, there is still a significant lack of understanding about the specific challenges faced by physiotherapists in Libya during the COVID-19 pandemic. This study aims to fill that gap by exploring Libyan physiotherapists’ experience with telerehabilitation during the COVID-19 pandemic, including their knowledge and readiness toward it. This research also identifies barriers and facilitators that will shape future practice in a post-pandemic context, shedding light on an area that has received little attention in telehealth research.

## 2. Methodology

This study employed a quantitative cross-sectional design, which involved collecting data through a self-administered questionnaire from participants at a specific moment. This approach allows us to get a clear picture of the participants’ experiences and perceptions regarding telerehabilitation, providing valuable insights into their current knowledge and practices.

### 2.1. Participants

A convenience sampling strategy was employed. While convenience sampling offers the advantage of being quick and cost-effective, it is important to acknowledge its limitations. Unfortunately, there is no published information about the number of physiotherapists in Libya, making it unfeasible to run a formal sample size calculation. While the use of convenience sampling limits generalizability and introduces potential selection bias, the sample size used in this study is still sufficient for statistical analysis. Due to limited time and resources, participants were selected based on their accessibility and willingness to participate. This allows for the identification of trends and barriers associated with telerehabilitation practice. A total of 122 physiotherapists were invited to participate in this study. Of these, 109 physiotherapists (54 males, 55 females; ages 25–55) completed and returned the questionnaire. The remaining 13 physiotherapists were excluded from the study due to either declining participation or failing to complete the questionnaire in full.

Participants in this study were required to have at least 1 year of experience as registered physiotherapists before the emergence of COVID-19, ensuring they had sufficient time in the field to provide informed insights. Exclusion criteria included physiotherapists who were not engaged in clinical practice, those who had been infected with COVID-19, and those who were absent during the distribution of the questionnaire. This helps to ensure that the findings are relevant and applicable to those directly affected by the pandemic’s restrictions. The study was conducted in accordance with the Declaration of Helsinki, and the protocol was approved by the Scientific Research and Ethics Committee at the University of Tripoli (SREC-UOT 02-2021), ensuring that the research adhered to ethical guidelines and standards for studies involving human participants.

### 2.2. Assessments

In June 2021, numerous public hospitals and private clinics in Tripoli, Libya, were contacted, but only five public hospitals and ten private clinics responded positively and agreed to allow us to collect data from their employees. The participating physiotherapists completed a self-administered questionnaire adapted from a published questionnaire applied to urologists [29]. To assess the reliability of this questionnaire, a test–retest reliability analysis was conducted, which yielded a coefficient of 0.81. This strong reliability score supports the use of the questionnaire as a consistent and dependable instrument for our study. Additionally, the level of agreement between the two sets of responses to gain further insight into the reliability of the questionnaire has been measured through telephone interviews with 12% of participants (13 out of 109). The result of the agreement demonstrates a high level of agreement (0.91), which indicates strong consistency in the questionnaire’s results. The original questionnaire was translated into Arabic and back-translated into English to ensure validity. All participants were fluent in Arabic, the final language of questionnaire administration.

Physiotherapists were approached in person to invite them to join our study. As part of this process, we provided them with an information sheet that explained the study’s purpose, emphasized that participation was completely voluntary, and outlined how much time it would take. Furthermore, for those who had more questions, we were happy to provide more details to help them understand the study better. Before they began filling out the questionnaire, we collected both verbal and written consent from all participants, making sure they met the inclusion criteria. This way, we ensured that everyone was well-informed and comfortable contributing to our research.

The questionnaire collected personal information and evaluated their knowledge of telerehabilitation. The questionnaire is designed to be concise yet comprehensive, and it includes two major parts. The personal information section included questions about age, sex, workplace, educational background, and whether their practice was affected by COVID-19, including reductions in workload, partial closures, or full closures. The second section began with a contingency question to evaluate participants’ familiarity with telerehabilitation. Those who indicated they were familiar with telerehabilitation then answered subsequent questions regarding its application in their practice, challenges encountered, commonly used telerehabilitation methods, duration of use, and compensation for telerehabilitation services. The questionnaire used a variety of scoring methods to understand how physiotherapists have engaged with telerehabilitation during the COVID-19 pandemic. It starts with demographic questions that categorize responses, while the impact of COVID-19 is measured with a simple binary scoring system. When it comes to knowledge of telerehabilitation, the same binary scoring approach is used. The duration of telerehabilitation use is scored on a scale, where longer experience earns higher points, and the assessment of the ability to receive payment for these services is also on a similar scale. Additionally, each type of telerehabilitation method used and the obstacles practitioners face are scored categorically.

### 2.3. Data Synthesis and Statistical Analysis

The data analysis was carried out by one of the coauthors with expertise in statistical methods using SPSS Statistics (Version 23.0; IBM Corp., Armonk, NY, USA). The data were encoded and analyzed, with descriptive statistics computed using frequencies and percentages for the assessed variables.

## 3. Results

### 3.1. Demographic Characteristics

All participants successfully completed and submitted the questionnaire. A total of 109 healthy physiotherapists participated, with ages ranging from 25 to 55 years. The demographic characteristics of the participants are summarized in Table 1, which includes frequency and percentage data on sex, age range, workplace, educational level, work experience, and current physical health status

### 3.2. Physiotherapists’ Knowledge About Telerehabilitation

**Figure 1:**
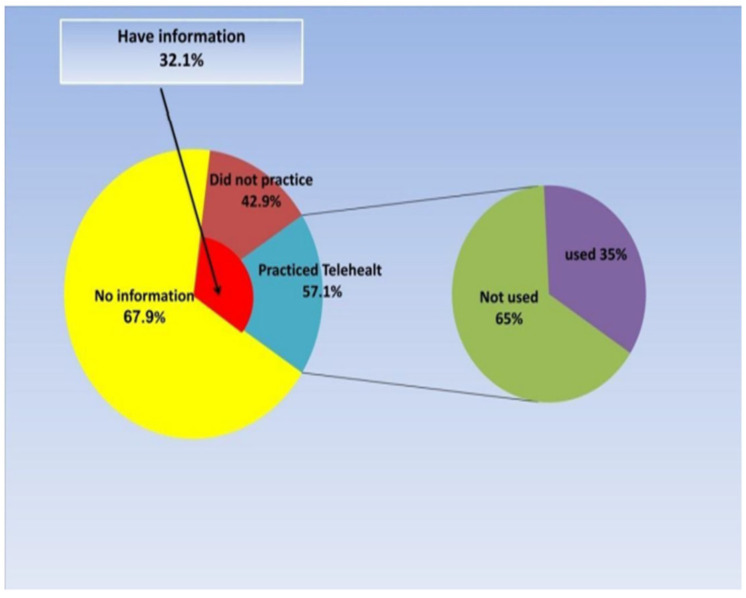
Information about physiotherapists and telehealth.

A significant majority (67.9%) of physiotherapists reported not knowing about telerehabilitation. Among those who were knowledgeable about telerehabilitation, only 57.1% had practiced telerehabilitation. Additionally, just 35% of those who practiced telerehabilitation have effectively integrated it into their physiotherapy practice, as shown in Figure 1. Table 2 provides details on the types of telerehabilitation utilized, duration of usage, payment structures for telerehabilitation services, and barriers that may hinder physiotherapists from adopting this technique.

## 4. Discussion

The COVID-19 pandemic has posed significant challenges for healthcare systems worldwide, affecting habits, economies, and health infrastructures. In Libya, the pandemic has fundamentally altered physiotherapy practices, with 64.2% of participating physiotherapists reporting that their work has been affected. This finding aligns with other research that indicates similar trends in countries with similar contexts; for instance, about 61% of Egyptian physiotherapists reported leaving their jobs during the pandemic [30]. Additionally, most Saudi physiotherapists (80%) reported disruption to their practice during the lockdown [31]. In contrast, developed countries with different healthcare systems reported lower frequencies of disruption. For example, MacDonald et al. reported that the public health orders and social restrictions led to only a 20.6% reduction in the utilization of physiotherapy services in Australia [25]. Moreover, they reported that Australian physiotherapists rated the importance of their involvement in managing patients with COVID-19 symptoms at a mean score of 50% (range 7–100%), while the necessity of isolating infected individuals from providers received a mean score of 79% (range 6–100%), indicating significant variability in responses [32]. We believe that when physiotherapists have a positive view of telehealth, they’re more motivated to seek out additional training and education. Research indicates that any form of telehealth education notably enhances the healthcare providers’ perceptions of usefulness, boosts their self-efficacy, increases their knowledge, and improves both their usage and satisfaction with telehealth [33], thereby reducing their knowledge gaps. This relationship suggests that fostering a positive perception of telehealth may be essential in encouraging ongoing professional development and overcoming barriers to effective implementation. These findings highlight the urgent need for Libyan healthcare systems to adapt physiotherapy practices in response to the challenges posed by the COVID-19 pandemic. Many physiotherapists have been affected, underscoring the importance of developing flexible care models and support systems to ensure the availability of services, especially in resource-limited contexts during future health crises.

Physiotherapists generally held a positive perception of telehealth services and were utilizing them before the COVID-19 pandemic [34]. While initial evidence indicates favorable views, during the pandemic, restrictions on telerehabilitation services for physiotherapists were eased, allowing for “e-visits” following advocacy from the APTA [26]. Research indicates that increased digital competence is associated with a greater likelihood of telehealth use [35]. Furthermore, telehealth training programs have been shown to increase the adoption of video visits [36,37]. As we move beyond the pandemic, integrating telerehabilitation has become vital in the post-pandemic era, as it provides ongoing support for individuals with chronic conditions and those who live in rural areas [38]. The pandemic revealed the need for training healthcare providers in telehealth practices [39] and building necessary infrastructure to support these services [40], alongside educating patients on how to use telehealth tools effectively, which can make a significant difference in their care experience [41]. Future research should focus on understanding the long-term outcomes and overall satisfaction of patients using telehealth services [39]. This is to ensure that telehealth delivers high-quality care and meets patients’ needs.

Our study found that 67.9% of physiotherapists were either unfamiliar with or uninformed about telerehabilitation, while only 32.1% had some knowledge of it. These findings are somewhat similar to results from other countries with comparable healthcare settings; for instance, 58.8% of physiotherapists working in Saudi Arabia reported that they had sufficient knowledge about telerehabilitation [42]. Furthermore, 57% of physiotherapists in Kuwait reported a lack of information about telerehabilitation [43], while 62% of physiotherapists in Malaysia reported that they had no prior experience with it [44]. In contrast, these findings differ from those in countries with different healthcare settings, such as Australia, where most physiotherapists had access to and could use telerehabilitation technologies [25]. This significant gap in knowledge and familiarity with telerehabilitation technologies in Libya, despite a generally positive perception of these services, suggests the need for implementation of educational and training programs that could raise awareness among physiotherapists about telerehabilitation and its effective utilization.

Furthermore, in this study, approximately 57% of those who are informed about telerehabilitation (which represents about 18% of the total sample) have effectively used telerehabilitation for patient management at some point in their career. However, 43% of these practitioners (which represent about 14% of the total sample) have not practiced it before. In Australia, physiotherapists reported that 84% of practitioners had access to telerehabilitation, with 96% able to use it; however, only 12% had utilized it before the pandemic [25]. Additionally, APTA noted a dramatic increase in physiotherapists providing live video consultations in the USA, rising from 2% before the pandemic to 48% a year later [26]. These findings highlight the importance and the need for developing targeted educational programs to help physiotherapists better understand and use telerehabilitation, especially in areas with low awareness levels. By improving knowledge and access to telehealth, we can make a real difference in patient care and outcomes, particularly in areas with limited resources and knowledge of telerehabilitation. The dramatic rise in live video consultations noted by the APTA shows the importance of targeted educational programs that can be adapted to different contexts. By fostering similar initiatives in Libya, we can enhance understanding and acceptance of telerehabilitation, even in resource-limited areas. Knowledge gained in the USA can inform best practices and facilitate partnerships that elevate telerehabilitation access in Libya, ultimately improving patient care and outcomes for everyone involved.

In this study, the predominant form of telerehabilitation utilized was internet video, followed by telephone calls, at rates of 62.5% and 50%, respectively. This is quite similar to findings from the American physiotherapists, where the APTA reported that over 70% of practitioners used online or smartphone platforms [26]. This suggests the growing familiarity with telerehabilitation, which could help in its wider adoption among physiotherapists and subsequently enhance access to the service, especially in remote areas.

It is important to identify barriers that could hinder the use of telerehabilitation and address them. In this study, the technology infrastructure factors are the significant barriers identified, namely, poor phone and internet coverage (71.6%), followed by illiteracy of the technology, either by the physiotherapist, patient, or both in using telerehabilitation technology (38.5%), where about 29% of participants reported high technology costs as a barrier. These results align with the literature where technology-related barriers, including inadequate access of the clients to telerehabilitation technology and inadequacy of providing technology by healthcare facilities, have been considered as the main technology barriers [26,45]. Similarly, lack of technological readiness was the top barrier to telerehabilitation use by Kuwaiti physiotherapists [43]. Illiteracy in using telehealth technology has also been mentioned in previous studies [45]. In Saudi Arabia, Aloyuni et al. reported that 23% of physiotherapists reported staff skills issues as a barrier to telerehabilitation use [41]. Further, a study in Malaysia has reported that patients either have difficulty using electronic devices or lack familiarity with social media platforms [44]. Furthermore, the high cost of telerehabilitation is considered one of the barriers that affect telerehabilitation implementation in Saudi Arabia [41]. It is essential to address the identified barriers, particularly concerning technology infrastructure and literacy, to effectively promote the use of telerehabilitation. This can be achieved by implementing targeted training programs for both physiotherapists and patients, which will enhance technological proficiency and ensure better access to telerehabilitation services. Additionally, improving internet coverage and reducing technology costs will be crucial steps in facilitating the adoption of telerehabilitation, which ultimately may lead to more equitable healthcare access in resource-limited settings. Although most of the participating physiotherapists in this study reported that they had attended educational workshops and training programs related to COVID-19 during the pandemic, none of these sessions specifically focused on telerehabilitation. Instead, the majority of the workshops concentrated on strategies for managing and treating patients affected by the virus, as well as on prevention measures against COVID-19. This lack of emphasis on telerehabilitation training highlights a gap in preparation for integrating virtual care into practice, which is reflected in the low level of knowledge among participants; approximately 68% of physiotherapists in Libya have no information or are unfamiliar with telerehabilitation. This gap is particularly concerning, given the increased demand for telerehabilitation services during the pandemic.

The study has several limitations, notably a small sample size of 109 physiotherapists, which may not adequately represent the wider physiotherapists population in Libya. Unfortunately, there is no published information about the number of physiotherapists in Libya; therefore, it was not feasible to run a formal sample size calculation. In spite of that, the study achieved a notable response rate of 89% among physiotherapists, indicating strong engagement and suggesting that the sample accurately represents the target population in Tripoli. The use of convenience sampling limits generalizability and introduces potential selection bias. Additionally, the sample was restricted to physiotherapists in Tripoli, which may not reflect the experiences of practitioners in other Libyan regions. Broader sampling across various cities is recommended for generalizability.

Moreover, the reliance on self-reported data could introduce bias, and excluding participants who had COVID-19 or were absent may result in missed insights. The cross-sectional nature of the study restricts the ability to observe changes in knowledge and attitudes over time. Furthermore, future studies could benefit from incorporating objective measurements and qualitative interviews for more robust data collection. Moreover, barriers such as poor internet access and high technology costs may not capture the full range of challenges faced by the Libyan physiotherapists.

### Implications of the Study

The insights from our study are vital as we continue to evolve telerehabilitation practices. We’ve identified some significant barriers, like limited knowledge about telerehabilitation, unreliable internet connections, and the high costs of technology. These findings highlight an urgent need for targeted training programs that can empower physiotherapists in Libya. It’s not just about teaching the technical side of telerehabilitation; we also need to focus on effective communication and ways to engage patients in a virtual setting. Moreover, our findings can help shape policy decisions that create supportive regulatory frameworks for integrating telerehabilitation into everyday physiotherapy practices. By addressing these issues, we can help transform physiotherapy in Libya, ensuring it meets the needs of patients in our changing world.

Looking ahead, the data collected during the pandemic offers a strong foundation for further exploring and adapting telerehabilitation. The challenges we have uncovered remind us of the importance of ongoing research and development in this field. As telehealth becomes a regular part of healthcare delivery, it’s crucial to promote strategies that effectively meet the needs of both practitioners and patients. Future studies should focus on tracking telerehabilitation outcomes over time, comparing its effectiveness to traditional in-person care. Moreover, encouraging a culture of innovation and adaptability within the physiotherapy profession could enhance service delivery, improve patient outcomes, and ultimately strengthen the healthcare systems in Libya and beyond.

## 5. Conclusions

Our study highlights the crucial role of telerehabilitation in the future of physiotherapy, emphasizing its value in connecting with patients, particularly those with chronic conditions or living in distant areas. The pandemic has taught us the importance of effective use, proper training for healthcare providers, and robust internet infrastructure to ensure access. Furthermore, embracing telerehabilitation as a core practice allows us to provide flexible care, using hybrid models that combine in-person visits with virtual check-ins. Educating patients on telerehabilitation tools empowers them to engage actively in their care. Moreover, promoting policies that support telerehabilitation initiatives certainly can improve the quality of service provided to patients and also can adjust the profession to meet the needs of patients in a changing and evolving healthcare environment.

## Figures and Tables

**Table 1:** Participants’ basic information.

		Frequency	%
Sex	Male	54	49.5
	Female	55	50.5
Age	<25 Years	25	22.9
	25–35 Years	47	43.1
	35–45 Years	30	27.5
	45–55 Years	7	06.4
Place of Work	Public	49	45.0
	Private	31	28.4
	Public + Private	29	26.6
Educational Level	Intermediate institute	8	07.3
	Higher institute	34	31.2
	Bachelors	59	54.1
	Master’s or PhD	8	07.4
Experience	<5 Yrs	41	37.6
	5–10 Yrs	27	24.8
	10–15 Yrs	16	14.7
	15–20 Yrs	15	13.8
	20–25 Yrs	8	07.3
	25–30 Yrs	1	00.9
	>30 Yrs	1	00.9
Physiotherapy Practice Affected by COVID-19	NOT affected	39	35.8
	Affected	70	64.2

**Table 2:** Telehealth information.

	Frequency	%	Cumulative %
Obstacles to using telehealth			
Illiteracy of PTs and patients in using telehealth	42		38.5
Bad phone & internet coverage	78		71.6
High prices of IT & a bad economy	32		29.4
Forms of telehealth used			
Recorded videos or messages	1		12.5
Telephone	4		50
Email	1		12.5
Smart phone	1		12.5
Internet video	5		62.5
Payment for telehealth			
No	4	50	
Yes, after COVID-19	2	25	
Yes, before COVID-19	2	25	
How long have you been using telehealth			
Since COVID-19	2	25	
<1 year	1	12.5	
1–2 years	1	12.5	
>2 years	4	50	

## Data Availability

Data are available upon request to the corresponding author.
